# Transcriptome analysis revealed growth phase-associated changes of a centenarian-originated probiotic *Bifidobacterium animalis* subsp*. lactis* A6

**DOI:** 10.1186/s12866-022-02474-5

**Published:** 2022-02-25

**Authors:** Hui Wang, Jieran An, Chengfei Fan, Zhengyuan Zhai, Hongxing Zhang, Yanling Hao

**Affiliations:** 1 Key Laboratory of Functional Dairy, Co-constructed by Ministry of Education and Beijing Municipality, College of Food Science and Nutritional Engineering, China Agricultural University, 17 Qing Hua East Road, Hai Dian District, Beijing, 100083 China; 2 Beijing Advanced Innovation Center for Food Nutrition and Human Health, College of Food Science and Nutritional Engineering, China Agricultural University, Beijing, China; 3 Department of Food Science, Beijing University of Agriculture, 7 Bei Nong Road, Changping District, Beijing, 102206 China

**Keywords:** *Bifidobacterium animalis* subsp. *lactis* A6, RNA-seq, Growth phase, Tad pili, Adhesion

## Abstract

**Background:**

The physiology and application characteristics of probiotics are closely associated with the growth phase. *Bifidobacterium animalis* subsp. *lactis* A6 is a promising probiotic strain isolated from the feces of a healthy centenarian in China. In this study, RNA-seq was carried out to investigate the metabolic mechanism between the exponential and the stationary phase in *B. lactis* A6.

**Results:**

Differential expression analysis showed that a total of 815 genes were significantly changed in the stationary phase compared to the exponential phase, which consisted of 399 up-regulated and 416 down-regulated genes. The results showed that the transport and metabolism of cellobiose, xylooligosaccharides and raffinose were enhanced at the stationary phase, which expanded carbon source utilizing profile to confront with glucose consumption. Meanwhile, genes involved in cysteine-cystathionine-cycle (CCC) pathway, glutamate dehydrogenase, branched-chain amino acids (BCAAs) biosynthesis, and Clp protease were all up-regulated in the stationary phase, which may enhance the acid tolerance of *B. lactis* A6 during stationary phase. Acid tolerance assay indicated that the survival rate of stationary phase cells was 51.07% after treatment by pH 3.0 for 2h, which was 730-fold higher than that of 0.07% with log phase cells. In addition, peptidoglycan biosynthesis was significantly repressed, which is comparable with the decreased growth rate during the stationary phase. Remarkably, a putative gene cluster encoding Tad pili was up-regulated by 6.5 to 12.1-fold, which is consistent with the significantly increased adhesion rate to mucin from 2.38% to 4.90% during the transition from the exponential phase to the stationary phase.

**Conclusions:**

This study reported growth phase-associated changes of *B. lactis* A6 during fermentation, including expanded carbon source utilizing profile, enhanced acid tolerance, and up-regulated Tad pili gene cluster responsible for bacterial adhesion in the stationary phase. These findings provide a novel insight into the growth phase associated characteristics in *B. lactis* A6 and provide valuable information for further application in the food industry.

**Supplementary Information:**

The online version contains supplementary material available at 10.1186/s12866-022-02474-5.

## Background

Probiotics are defined as “live microorganisms which when administered in adequate amounts confer a health benefit on the host” [1]. Some probiotics confer a number of health-promoting benefits to human host, such as competitive exclusion of pathogenic bacteria, modulation of immune system and enhancement of epithelial barrier function [2–4]. Therefore, probiotics, mainly including lactobacilli and bifidobacteria, have been widely applied as food and dietary supplements [5]. However, the physiology and application characteristics of probiotics are closely associated with the growth phase. After spray drying, the viability of *Lactobacillus rhamnosus* GG was approximately 20 times higher at stationary phase than that of cells from the exponential phase [6]. Meanwhile, the maximum adhesion ability of *L. rhamnosus* GG to Caco-2 cells at the early stationary phase was ten times higher than that of cells at the exponential phase [7]. Therefore, it is noteworthy to investigate the growth phase-associated metabolic mechanisms in probiotics.

Omics techniques provide an effective strategy to reveal growth phase-associated metabolic mechanisms. Transcriptomic analysis revealed a shift from the cell division gene expression at the lag phase to a carbohydrate metabolism-related gene repertoire at the exponential growth phase in *Bifidobacterium bifidum* PRL2010 [8]. Meanwhile, transcriptomic analysis of *Lactobacillus casei* Zhang showed that genes involved in carbohydrate metabolism, inorganic ion transport, and chaperones were highly expressed at the stationary phase, whereas genes related to nucleotide transport and metabolism, energy production and conversion were predominantly expressed during the exponential phase [9]. Furthermore, combined transcriptomic and proteomic analyses demonstrated that the shift from glucose fermentation to galactose utilization and the transition from homolactic to mixed acid fermentation were observed from the exponential to the stationary growth phase in *L. rhamnosus* GG [10].

*Bifidobacterium animalis* subsp. *lactis* A6 (*B. lactis* A6) was isolated from a healthy centenarian in the Bama County of the Guangxi Zhuang Autonomous Region in China [11], which is famous for having a population with a high life-expectancy. Previous study showed that *B. lactis* A6 had high acid resistance to low pH and could improve obesity in mice [12, 13]. Unlike DNA microarray method which is constrained by the certain levels of background signal and the limited dynamic detection range, RNA-Seq, as the Next-Generation Sequencing technology, provides higher efficiency and sensitivity for microbial transcriptomic analysis [14]. In this study, RNA-seq was performed to investigate the growth phase-related changes between the exponential and stationary phase in *B. lactis* A6. Our results will provide new insights for the rational application of *B. lactis* A6 as probiotics.

## Results

### Growth of *B. lactis* A6 strain

**Fig. 1:**
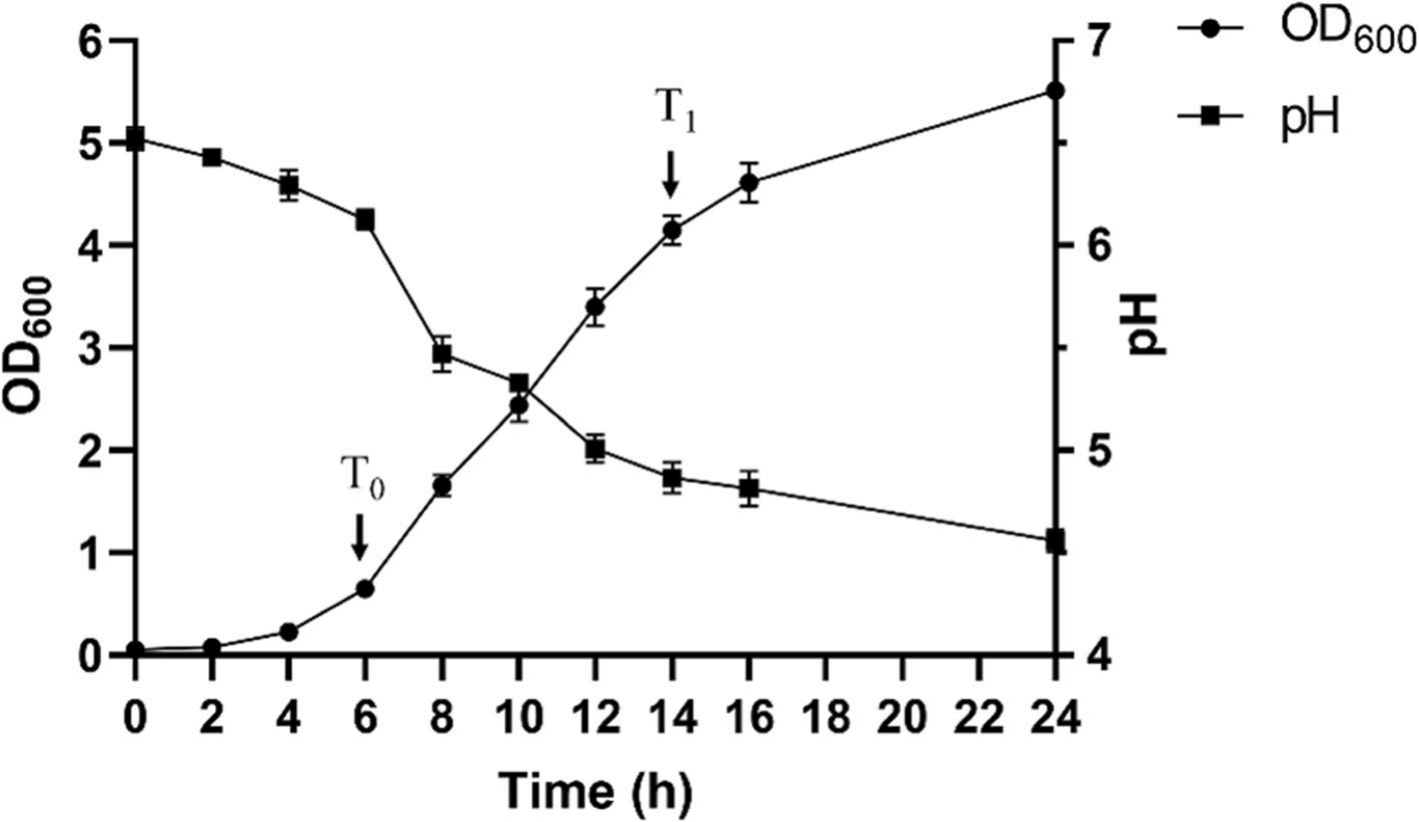
Growth of *B. lactis* A6 strain in MRSc broth incubated at 37°C for 24 h. The OD_600_ and pH were determined from 0 to 24 h. *B. lactis* A6 grew up to an average OD_600_ around 0.6~0.8 was collected as the exponential phase samples (time point T_0_), while OD_600_ around 4.0~4.2 was collected as the stationary phase samples (time point T_1_).

In order to investigate the changes of growth situation, *B. lactis* A6 was cultured in 15 ml de Man-Rogosa-Sharp broth supplemented with 0.05% (w/v) L-cysteine (MRSc) for 24 h. As shown in Fig. 1, the OD_600_ value reached 5.4 at the end-point of fermentation at 24 h, and the pH was reduced to 4.6 from an initial value of 6.5. The bacteria entered into the exponential growth phase at time point T_0_ with OD_600_ ~ 0.6 and the stationary growth phase at time point T_1_ with OD_600_ ~ 4.0.

### Global gene expression profiles in the stationary phase compared to the exponential phase

**Fig. 2:**
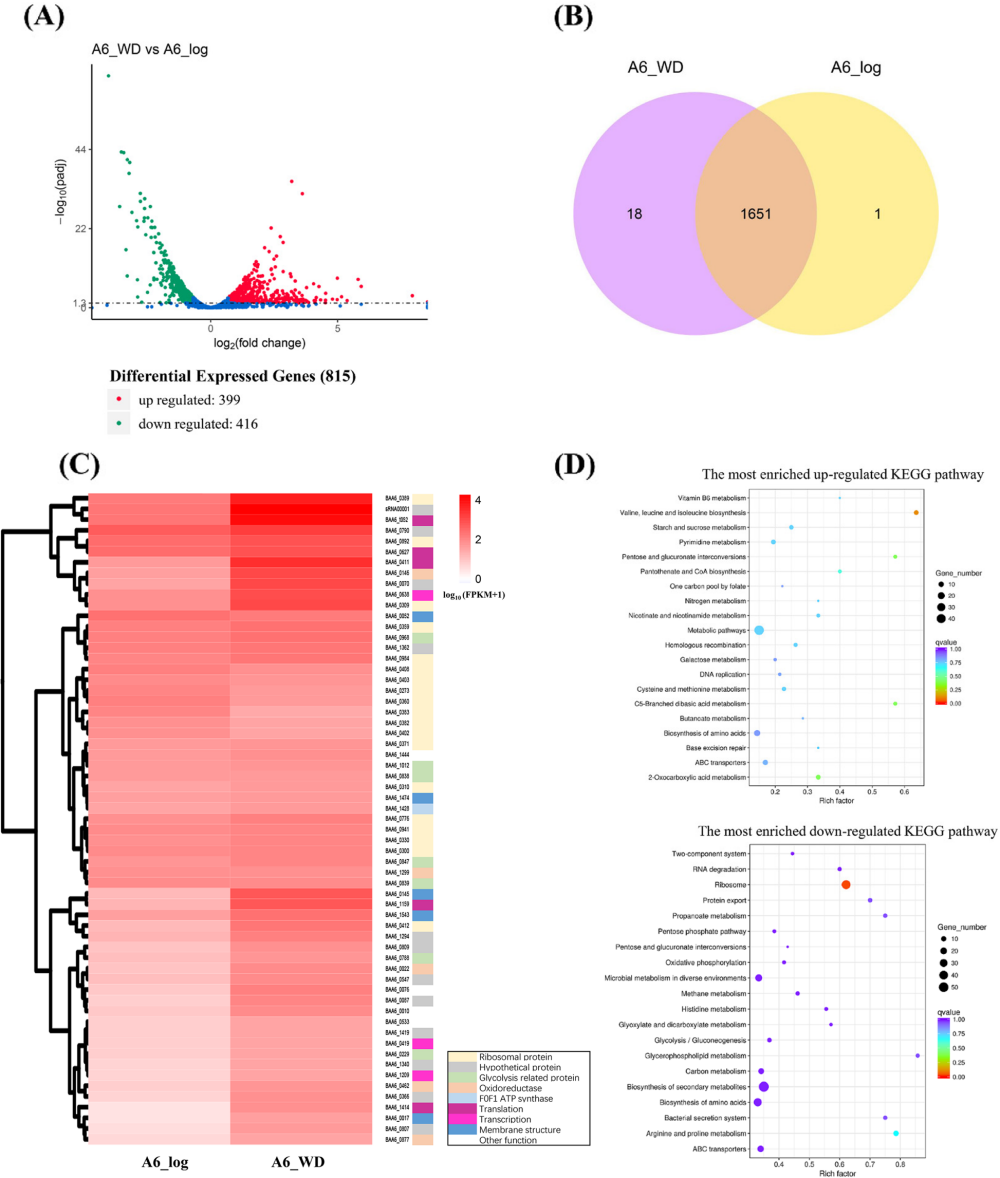
RNA-seq analysis of DEGs. (**A**) Volcano plot of DEGs during the stationary phase (A6_WD) compared to the log phase (A6_log). The red plots represent up-regulated genes, green plots represent down-regulated genes. (**B**) Venn diagram of expressed genes based on FPKM value > 1. The overlapped region represents co-expressed genes both in the stationary and log phase, while non-overlapped area represents the specifically expressed genes in each growth phase. (**C**) Hierarchical clustering analysis of top expressed genes both in the log and stationary growth phase. The depth of color indicates the expression level of genes calculated by log_10_(FPKM+1). The biological process for each gene is summarized right behind gene number. (**D**) Scatter plot of enriched KEGG pathway of DEGs in the stationary phase compared to the log phase. The size of black circle indicates the numbers of DEGs involved in each biological pathway, and the color of points correspond to different q-value ranges.

The number of clean reads and the reads mapping to the reference genome are shown in Table S1 and S2. After removing low quality reads (a single sequencing read contains more than 50% bases with Phred Quality Score ≤ 20) and adaptor sequences, more than 19.6 million high-quality reads per sample were generated. The clean reads were aligned to the whole reference genome sequence, and more than 99% of the clean reads for each sample were mapped to the genome. Correlation of gene expression level between three independent biological replicates was shown by the Pearson’s correlation coefficient, which was more than 0.872 for each group (Fig. S1). These results indicated the obtained transcriptome data were suitable for further analysis. Differential expression analysis showed that a total of 815 differential expression genes (DEGs) consisted of 399 up-regulated and 416 down-regulated genes in the stationary phase (A6_WD) compared to the exponential phase (A6_log) (Fig. 2A). The Veen diagram showed 1651 co-expressed genes (FPKM value > 1) both in the stationary and log phase, 18 specifically expressed genes (SEGs) in the stationary phase, and 1 SEG in the log phase (Fig. 2B). Among these co-expressed genes, ~ 60 top expressed genes are summarized in Fig. 2C, which are mainly involved in the core metabolic pathways, including transcription and translation (ribosomal protein), energy production (ATP synthase and glycolysis), and redox reaction (oxidoreductase). The SEGs in each growth phase are listed in Table S3, however, most of these genes encoded hypothetical proteins. The KEGG pathway enrichment analysis of A6_WD compared to A6_log was summarized separately in Fig. 2D. It revealed that the up-regulated genes are mainly participated in the branched-chain amino acids biosynthesis, ABC transporters and metabolic pathways, while down-regulated genes are associated with ribosome, secondary metabolites biosynthesis, and carbon metabolism (glycolysis pathway). Moreover, a list of predicted promoters of constitutively or specifically expressed genes in both growth phases was summarized in the Supplemental profile. Based on the KEGG pathway analysis, the DEGs involved in carbohydrate uptake and metabolism, oligopeptides uptake, peptidoglycan biosynthesis, acid tolerance resistance and adhesion to mucin were discussed in detail as follows.

### Carbon source utilization profile was expanded during the stationary phase

**Fig. 3:**
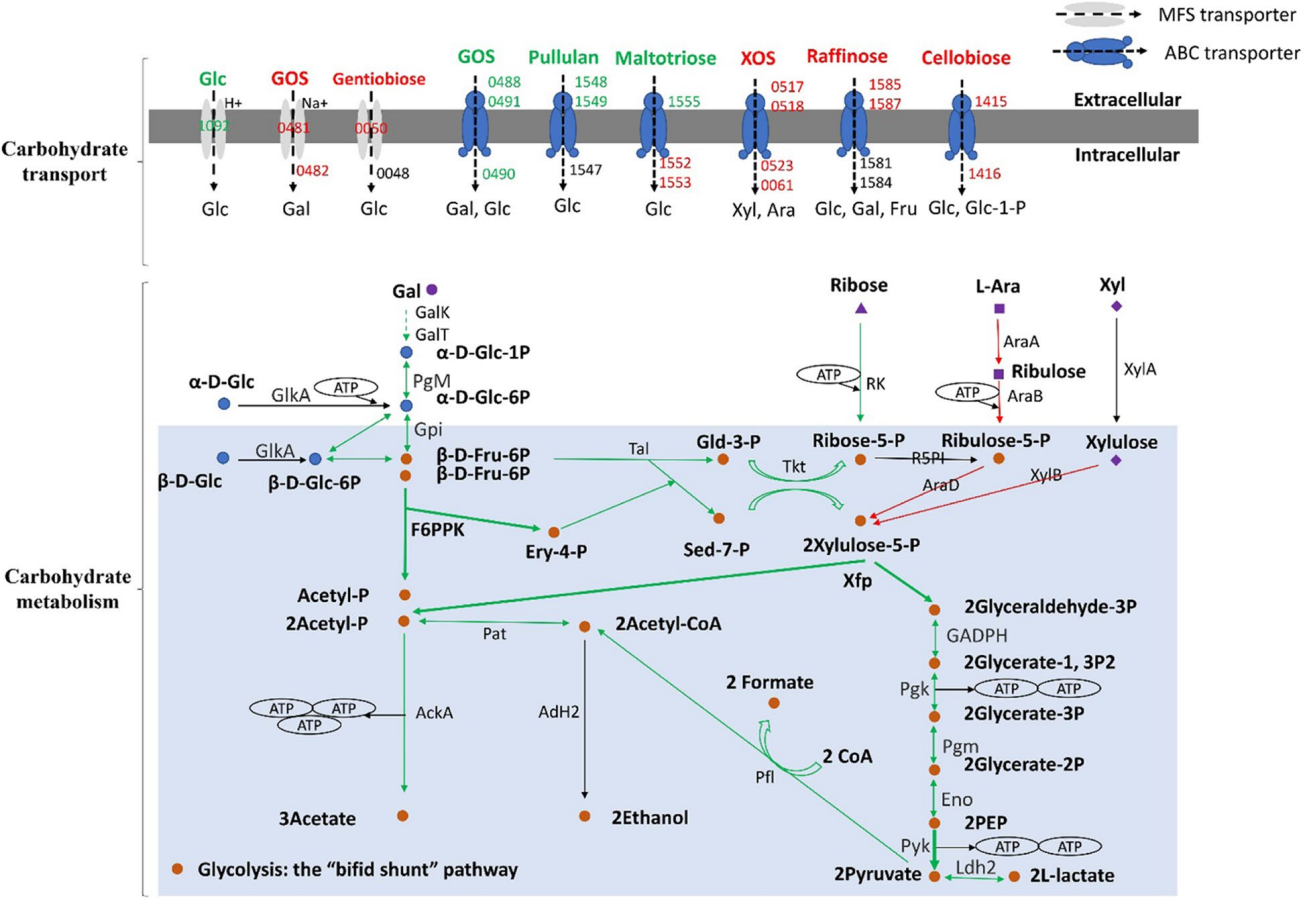
Graphical representation of DEGs involved in carbohydrate transport and metabolism in *B*. *lactis* A6. Arrows or gene numbers in black, red or green color show genes which are unchanged, up-regulated or down-regulated at transcriptional level in the stationary phase compared to the log phase, respectively. GOS, galactooligosaccharides; XOS, xylooligosaccharides; Glc, glucose; Gal, galactose; Xyl, xylose; Ara, L-arabinose; Fru, fructose; GalK, galactose kinase; GalT, galactose-1-phosphate uridylyltransferase; Glc-1-P, glucose-1-phosphate; Glc-6-P, glucose-6-phosphate; GlkA, glucokinase; PgM, phosphoglucomutase; Gpi, glucose-6-phosphate isomerase; β-D-Fru-6P, β-D-fructose-6-phosphate; F6PPK/Xfp, fructose-6-phosphate/Xylulose-5-phosphate phosphoketolase; Ery-4-P, erythrose-4-phosphate; Acetyl-P: acetyl-phosphate; Tal, transaldolase; Gld-3-P, glyceraldehyde 3-phosphate; Sed-7-P, sedoheptulose 7-phosphate; Tkt, transketolase; R5PI, ribose-5-phosphate isomerase; RK, ribose kinase; AraA: L-arabinose isomerase; AraB: L-ribulokinase; AraD: L-ribulose-5-phosphate 4-epimerase; XylA, xylose isomerase; XylB, xylulokinase; GADPH, glyceraldehyde 3-phosphate dehydrogenase; Pgk, phosphoglycerate kinase; Pgm, phosphoglyceromutase; Eno, enolase; PEP, phosphoenolpyruvate; Pyk, pyruvate kinase; Ldh2, lactate dehydrogenase; Pfl, formate acetyltransferase; Pat, phosphate acetyltransferase; AckA, acetate kinase; Adh2, bifunctional acetaldehyde-CoA/alcohol dehydrogenase.

In this study, genes *BAA6_0517-0518* and *BAA6_1585-1587* encoding two ABC-type transporters for xylooligosaccharides (XOS) and raffinose transport were up-regulated over 2.0-fold during the stationary phase, however, genes *BAA6_0488-0491*, *BAA6_1555*, and *BAA6_1548-1549* encoding three ABC-type transporters for galactooligosaccharides (GOS), maltotriose, and pullulan transport were down-regulated over 1.8-fold. Interestingly, *BAA6_1415* encoding a putative cellobiose ABC transporter permease protein CebG and *BAA6_1416* encoding cellobiose phosphorylase CbpA were found simultaneously up-regulated over 3.6-fold in the stationary phase. Exogenous cellobiose is transported into the bacterial cell by CebG, and then catalyzed by CbpA to yield one molecule of glucose-1-phosphate and glucose [15]. Moreover, genes *BAA6_0481* and *BAA6_0050* encoding two putative MFS transporters for the uptake of GOS and gentiobiose were up-regulated 6.2 and 3.6-fold, respectively (Table S4 and Fig. 3). A phenomenon of selectively expressed genes involved in uptake of exogenous carbon sources was observed during transition from the exponential phase to the stationary phase in *B. lactis* A6.

For glucose transport and metabolism, gene *BAA6_1092* encoding a putative glucose uptake permease was down-regulated 6.1-fold during the stationary phase. Meanwhile, eighteen genes involved in glucose metabolism called “the bifid shunt” were also down-regulated 1.5~3.6-fold during the stationary phase (Table S4 and Fig. 3). Among them, gene *BAA6_0968* encoding a bifunctional fructose-6-phosphate/xylulose-5-phosphate phosphoketolase (F6PPK/Xfp) was decreased 1.7-fold, which catalyzes the conversion of xylulose 5-phosphate or fructose 6-phosphate to acetyl phosphate [16]. Furthermore, gene *pyk*, *ldh2*, *pfl*, and *ackA* encoding pyruvate kinase, lactate dehydrogenase, formate acetyltransferase and acetate kinase were down-regulated 1.5, 2.6, 3.6 and 1.7-fold, respectively, which are involved in the production of pyruvate, L-lactate, formate and acetate [17]. Remarkably, genes *araA*, *araB*, and *araD* for L-arabinose metabolism to xylulose-5-P and gene *xylB* for xylulose metabolism to xylulose-5-phosphate were all up-regulated over 2.0-fold at the stationary phase [18]. These results showed that the glycolysis pathway was slowed down during the stationary phase with glucose consumption, but *B. lactis* A6 tends to increase gene expression for selective utilization of exogeneous carbon sources, such as XOS, cellobiose, and L-arabinose.

### Oligopeptides uptake was enhanced to adapt to changes in growth

Genes *aspC*, *metE*, *pheA*, *argFGH*, *proC*, and *glnA* involved in aspartate, methionine, tyrosine, phenylalanine, arginine, proline, and glutamine biosynthesis were down-regulated 1.6~16.4-fold during the stationary phase (Table S4). Meanwhile, genes *BAA6_1269-1270*, *BAA6_0680-0683*, and *BAA6_1082-1083* encoding three pairs of ABC-type amino acid transporters for aspartate, glutamate and methionine uptake were also down-regulated 2.0~4.5-fold. However, the *opp* operon, *BAA6_0566-0570*, encoding an ABC oligopeptide transporter OppABCDF was upregulated 2.0~5.2-fold, which are involved in the uptake of oligopeptides for bacteria [19]. Simultaneously, genes *dppD* and *dppC* encoding two components of dipeptide transporter DppABCDF complex were increased 8.4 and 5.8-fold, respectively. DppABCDF is specialized in dipeptide transportation to support bacterial growth in *Escherichia coli* [20]. Remarkably, genes *BAA6_0186* and *BAA6_0230* encoding two dipeptidases were all up-regulated over 1.7-fold. Dipeptidase catalyzes the hydrolysis of dipeptides to produce free amino acids for bacterial growth [21]. Therefore, *B. lactis* A6 tends to utilize exogenous amino acids by oligopeptides uptake, rather than *de novo* synthesis of single amino acid during the stationary phase.

### Peptidoglycan biosynthesis were repressed in the stationary phase

Gene *BAA6_0562* encoding glutamine-fructose-6-phosphate transaminase was down-regulated 2.2-fold during the stationary phase, which catalyzes the first step of peptidoglycan biosynthesis from fructose-6P (Fig. S2). The formed glucosamine-6-phosphate was then catalyzed by phosphoglucosamine mutase encoded by *BAA6_1325* to glucosamine-1-phosphate (GlcN-1P). The gene *glmU* encoding a bifunctional protein GlmU was also down-regulated 3.1-fold, which catalyzes the biosynthesis of UDP-N-acetylglucosamine from GlcN-1P [22]. Additionally, *murC*, *murD*, and *murF* were down-regulated over 1.5-fold, which are involved in the addition of L-alanine, D-glutamate, and D-alanyl-D-alanine to UDP-N-acetylmuramate to form the skeletal structure of a DAP-type peptidoglycan. Moreover, gene *BAA6_0083* encoding transpeptidase (DD-TPase) and *murJ* encoding a putative peptidoglycan lipid II flippase were also down-regulated over 1.6-fold. DD-TPase was reported to synthesize cross-linked peptidoglycan from lipid intermediates, and then the lipid-linked peptidoglycan precursors were transported from the inner cytoplasmic membrane to the outer leaflet by flippase during cell wall formation [23, 24]. Furthermore, gene *BAA6_0084* encoding a bacterial cell division membrane protein and genes *BAA6_0550*, *BAA6_1198*, and *BAA6_1201* encoding cell division protein FtsX, FtsQ, and FtsW were all down-regulated around 2.0~3.6-fold during the stationary phase (Table S4). Taken all together, genes involved in peptidoglycan biosynthesis, a key cell wall component in bifidobacteria, and cell division were generally repressed during the stationary phase in *B. lactis* A6, reflecting a retarded growth situation at the stationary growth phase.

### Acid tolerance response

Gene *BAA6_0572* encoding cystathionine beta-synthase (CysK) and *BAA6_0947* encoding cystathionine beta-lyase (MetC) were upregulated 7.1 and 2.8-fold, respectively. (Table S4). CysK catalyzes the synthesis of cystathionine from homocysteine and serine, and MetC catalyzes the cleavage of cystathionine to homocysteine, pyruvate and NH_3_, which constitute the cysteine-cystathionine-cycle (CCC) in response to acid stress [25]. Meanwhile, gene *serB* encoding phosphoserine phosphatase for the biosynthesis of serine was also up-regulated 1.8-fold during the stationary phase. Moreover, gene *BAA6_0010* encoding a NADP-specific glutamate dehydrogenase (GdhA) was up-regulated 3.2-fold, which catalyzes glutamate to 2-oxoglutarate and NH_3_. In contrast, gene *BAA6_0708* and *BAA6_1220* encoding glutamine synthetase 1 and 2 were respectively down-regulated 7.4- and 2.2-fold, which are involved in catalyzing the biosynthesis of glutamine from glutamate and NH_3_. In addition, gene *livK* encoding a substrate-binding protein for the transport of branched-chain amino acids was up-regulated 3.1-fold during the stationary phase. Meanwhile, *BAA6_0283* encoding acetolactate synthase and *BAA6_0146* encoding ketol-acid reductoisomerase were also up-regulated 2.6- and 4.1-fold, respectively. Acetolactate synthase catalyzes the biosynthesis of acetolactate and 2-aceto-2-hydroxybutanoate from pyruvate, which are further catalyzed by ketol-acid reductoisomerase for valine, leucine and isoleucine biosynthesis [26]. Meanwhile, *BAA6_0675* encoding ATP-binding subunit ClpE and *BAA6_1072*~1073 encoding two Clp protease proteolytic subunits ClpP1 and ClpP2 were also up-regulated 1.6, 1.6 and 3.3-fold, respectively (Table S4). Thus, genes involved in cysteine-cystathionine-cycle (CCC) pathway, glutamate dehydrogenase, branched-chain amino acids (BCAAs) biosynthesis, and Clp protease were all up-regulated in the stationary phase, which may contribute to enhance acid tolerance of *B. lactis* A6 during fermentation.

**Fig. 4:**
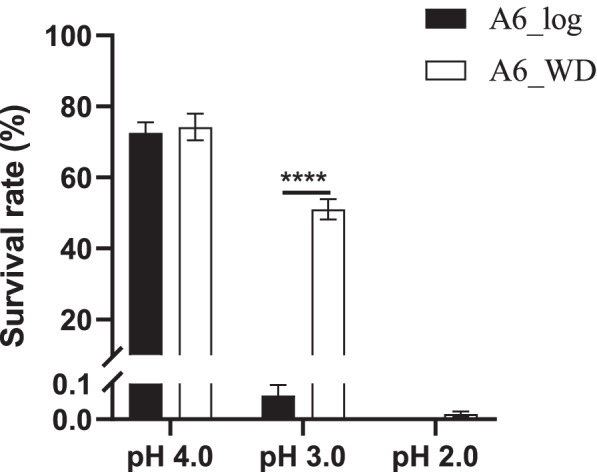
Survival rate changes of *B. lactis* A6 after acid treatment. Average of ~10^8^ CFU/mL bacterial cells during the log growth phase (A6_log) or the stationary growth phase (A6_WD) were treated with pH 4.0, 3.0, 2.0 for 2 h, respectively. Values are shown as means ± stand deviation (SD). Statistically significant differences between A6_log and A6_WD were identified using unpaired Student's t-test. (n=3; ****, *P* < 0.0001).

To investigate the general acid tolerance of *B. lactis* A6 under the log and stationary growth phase, an average of ~10^8^ CFU/mL bacterial cells of each growth phase were treated with acidic pH 4.0, 3.0, 2.0 for 2 h, respectively. As shown in Fig. 4, no significant difference was observed in survival rate between the two growth phases after treatment with pH 4.0 for 2 h. However, the survival rate of log phase cells was dramatically decreased to 0.07% after treatment with pH 3.0 for 2 h, significantly lower than that of 51.07% with stationary phase cells. No viable cells were detectable in log phase cells after treatment with pH 2.0 for 2 h, while ~5 log CFU/mL decrease of viable cells was observed in stationary phase cells from 8.86 to 3.99 log CFU/mL and the survival rate was 0.002%. These results suggested *B. lactis* A6 cells in stationary phase are more acid-tolerant than the log phase.

### Adherence to mucin was enhanced during the stationary phase

**Fig. 5:**
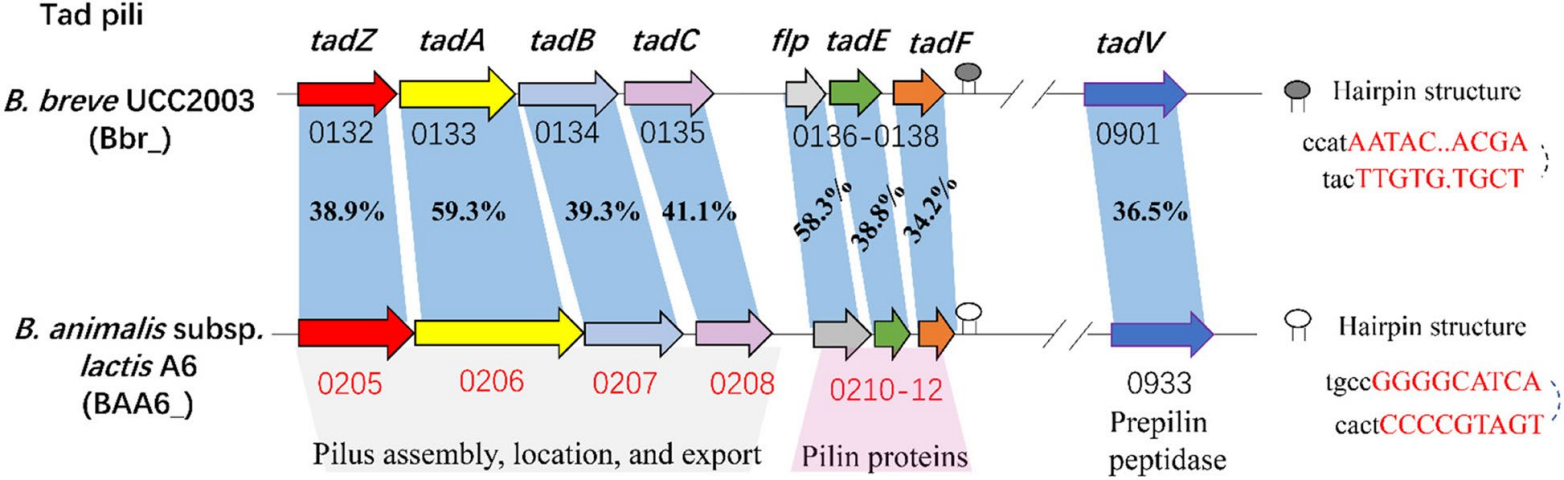
Schematic representation of the tad locus involved in pilus biosynthesis of *B. lactis* A6 compared with *B. breve* UCC2003. Each arrow represents an open reading frame (ORF), and the size of which is proportional to the length of the arrow. Gene name or gene number is right above or below the arrow. Coloring of the arrows represents the different function of the gene as indicated above each arrow. For *B. lactis* A6, gene numbers in red or black indicate transcriptionally up-regulated or unchanged genes during the stationary phase compared to the log phase. The amino acid identity of the relevant encoded proteins is indicated in percentages. The predicted transcription terminator with a hairpin structure is indicated by the stem loop.

**Fig. 6:**
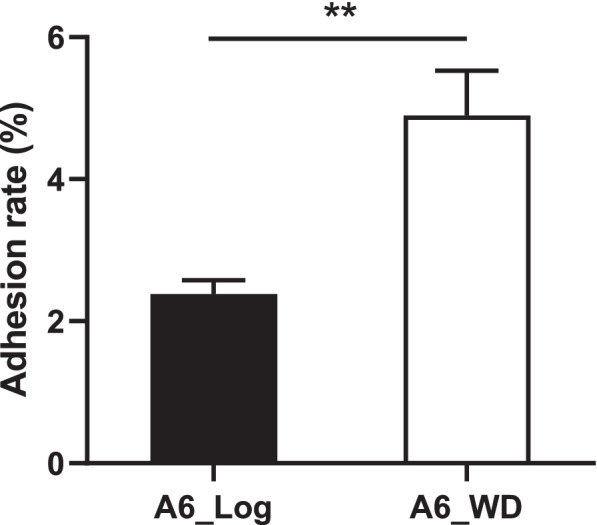
Adhesion to mucin of *B. lactis* A6. Statistically significant differences between the exponential phase (A6_log) and the stationary phase (A6_WD) were identified using an unpaired Student's t-test. (*n*=3; **, *p* < 0.01).

In this study, a putative gene cluster of *BAA6_0205~0212* involved in type IVb tight adherence (Tad) pili biosynthesis was found up-regulated 7.0~12.4-fold during the stationary phase compared to the log phase (Table S4). Among them, genes *flp, tadE and tadF* encoding a fimbrial protein prepilin and two pseudopilins were upregulated 7.5, 7.7 and 8.0-fold, respectively, which are participated in prepilin proteins biosynthesis. Meanwhile, gene *tadV* encoding a prepilin peptidase was up-regulated 1.5-fold in the stationary phase. Prepilin peptidase catalyzes the formation of mature pilin from prepilin proteins [27]. In addition, *tadZ* and *tadABC* encoding four Flp pilus assembly proteins involved in pilus assembly and localization were also up-regulated 10.4, 7.0, 12.4 and 10.6-fold, respectively. A gene cluster - “*tad*_*2003*_” locus (*Bbr_0132~0138* and *Bbr_0901*) encoding Tad pili has been reported in *Bifidobacterium breve* UCC2003 (GenBank accession number NC_020517.1), which are responsible for colonization in murine gut [28]. The amino acid sequence alignment of Tad pili between *B. breve* UCC2003 and *B. lactis* A6 showed an amino acid sequence identity among 34.2%-59.3% (Table S5 and Fig. 5). Tad pili is an important adhesive structure identified in bifidobacteria [29], and the adhesion of *B. lactis* A6 to mucin showed that the adhesion rate was 4.90% for stationary cells, significantly higher than that of 2.38% for exponential cells (Fig. 6). These findings further confirmed the adhesion to mucin was expressed in a growth phase-dependent manner in *B. lactis* A6 strain, and the enhanced expression of Tad pili may facilitate its colonization and maintenance in the intestine of host receptor.

## Discussion

Probiotics are widely accepted to be beneficial for the maintenance of the gut homeostasis, improvement of the immune system, and amelioration of various metabolic disease [30, 31]. In recent years, the consumption of probiotics to promote health has grown rapidly worldwide and become an independent industry [32]. Notably, the physiology and application characteristics of probiotics are closely associated with the growth phase. Many studies have focused on the overall growth phase-associated changes during fermentation based on transcriptome and/or proteome technology among *L. casei*, *L. rhamnosus*, and *B. longum* [9, 10, 33]. For the species of *B. lactis*, transcriptomic techniques have been applied to reveal the oligosaccharides uptake and metabolism in *B. lactis* Bl-04 [15], the xylo-oligosaccharides utilization in *B. lactis* BB12 [34], and the tetracycline response in *B. lactis* strains Bl-04 and HN019 [35]. However, to the best of our knowledge, no transcriptomic study about the growth phase-associated changes has been reported in this species. Andersen and colleagues identified the genetic loci involved in the uptake and catabolism of 11 potential prebiotics oligosaccharides based on transcriptional analysis in *B. lactis* Bl-04. The results showed that *B. lactis* Bl-04 exhibits extensive capabilities to utilize indigestible carbohydrates in the human gastrointestinal tract. For cellobiose metabolism, genes *Balac_1569~1570* encoding two permease components and *Balac_1572* encoding oligosaccharide-binding protein of an ABC transporter responsible for cellobiose uptake were found significantly up-regulated when cellobiose was used as carbon source compared with glucose. However, in the present study, we observed two genes *BAA6_1415* encoding cellobiose ABC transporter permease protein CebG and *BAA6_1416* encoding cellobiose phosphorylase CbpA were increased 3.6 and 7.7-fold in the stationary phase compared with the log phase, respectively. *B. lactis* A6 tends to increase gene expression for utilization of exogeneous cellobiose with glucose consumption in the stationary growth phase. The strain of *B. lactis* A6 was isolated from the feces of a Bama centenarian, whose diet was rich in plant-based fibers [36]. Plant derived carbohydrates, such as cellulose, could be catalyzed to produce soluble cellobiose by cellobiohydrolases in the human gut [37]. Therefore, the up-regulation of cellobiose transport and utilization may contribute to the adaptation of *B. lactis* A6 in the intestine of healthy centenarians.

Peptidoglycan is the major component of Gram-positive bacterial cell wall and is essential for the maintenance of bacterial integrity and shape [38]. In this study, most of genes involved in peptidoglycan biosynthesis and cell division were down-regulated in the stationary phase compared with the log phase, which suggested that the bacterial growth was generally repressed during this stage. Laakso et al. has been reported the growth phase-related changes of *L. rhamnosus* GG when grew in industrial-type whey medium, and the results showed that two genes *murC* and *murD* involved in peptidoglycan biosynthesis were significantly down-regulated both at the transcriptomic and proteomic level in the stationary growth phase compared to the mid-log phase [10]. This result is consistent with what has been observed in our study. Interestingly, genes involved in oligopeptides uptake and dipeptides metabolism were found up-regulated in the stationary phase. In contrast, genes involved in *de novo* synthesis of amino acid, such as aspartate, methionine, tyrosine, phenylalanine, arginine, proline, and glutamine were highly down-regulated. It has been reported that lactic acid bacteria could lower the synthesis of DNA and proteins and shift physiological state to the stationary phase due to carbohydrate starvation conditions during fermentation [39]. At this stage, an increased catabolism of carbon sources like peptides and free amino acids was observed for energy production [40]. Therefore, the up-regulated oligopeptides uptake and dipeptides metabolism may produce more free amino acids for energy production in bacterial cells of *B. lactis* A6 and then elaborate a potential survival strategy to adapt to glucose consumption during growth.

For acid tolerance response, *B. lactis* A6 exhibited various strategies to regulate the pH reduction during growth. NH_3_ could neutralize intracellular excess H^+^ to respond acid tolerance in bacteria. Three metabolic pathways have been reported with NH_3_ production: cysteine-cystathionine-cycle (CCC) pathway and deamination of glutamate and BCAAs. In *B. longum* BBMN68, three genes of *malY1*, *metC3*, and *cysK* involved in CCC pathway were up-regulated 9.25-, 2.71-, and 2.84-fold after acid treatment, respectively [25]. Glutamate dehydrogenase (GDH) catalyzes the reversible oxidative deamination of glutamate to α-ketoglutarate and NH_3_. Under low pH conditions, this reaction towards to produce more NH_3_ [41]. In addition, the overexpressed BCAAs biosynthesis has been reported in an acid-pH-resistant mutant *B. longum* 8809dpH after growth at an acidic condition. A higher intracellular NH_4_^+^ concentration was observed to respond acid treatment, which are positively associated with the deamination of BCAAs [42]. In this study, genes involved in the CCC pathway (*cysK* and *metC*), glutamate dehydrogenase (*gdhA*) and BCAAs biosynthesis (*ilvB* and *ilvC*) were all up-regulated over 2.6-fold in the stationary phase compared to the log phase, which may contribute to NH_3_ production for acid tolerance response in *B. lactis* A6. Moreover, Clp protease has been reported for participating general stress responses. Clp proteases could recognizes and degrades irreversibly damaged proteins in response to acid stress [25]. The up-regulated Clp proteases for degradation of damaged proteins could facilitate amino acids recycling in *B. lactis* A6 under acid stress. Furthermore, the general acid tolerance between the log phase and stationary phase cells has been verified by *in vitro* experiment, the results showed that the stationary cells are more acid-tolerant than the log cells in *B. lactis* A6.

Colonization in the mammalian gastrointestinal tract by microbes is believed to play an essential part to confer health benefits [43]. A Tad pili encoding gene cluster - “*tad*_*2003*_” locus was identified in *B. breve* UCC2003, which was proved to be essential for colonization and persistence in murine gut as a conserved host-colonization factor [28]. The “*tad*_*2003*_” locus encodes eight proteins for the biosynthesis of a mature Tad pilus. TadE and TadF are participated in the prepilin precursor biosynthesis, and TadA is an ATPase to provide energy for Flp-pilus assembly. TadB and TadC are thought to channel the energy of ATP hydrolysis into Flp-pilus polymerization or serve as an inner membrane scaffold for the assembly of the pilus biogenesis apparatus. Then the Tad apparatus is directed to the cell poles by TadZ [27, 44]. In this study, a putative Tad pili encoding gene cluster *BAA6_0205 ~ 0212* was highly expressed during the stationary phase, which exhibited similar gene cluster structure and higher amino acid identity of 34.2%-59.3% with “*tad*_*2003*_” locus. In addition, Tad pili could bind to carbohydrate moieties present in glycoproteins or glycolipids receptors of mucus layer, which covered on the surface of intestinal epithelial cells [45, 46]. Thus, the enhanced expression of Tad pili in *B. lactis* A6 strain during the stationary phase may facilitate its colonization and maintenance in the intestine of the host. To verify the hypothesis, the adhesion rate of *B. lactis* A6 to mucin was performed *in vitro* with the log and stationary phase bacterial cells, respectively. A significantly increased adhesion rate to mucin from 2.38% to 4.90% was observed during the transition from the exponential phase to the stationary phase.

## Conclusions

Probiotic bacteria harvested at different phases of growth has been shown to cause different application characteristics and mucosal responses in human. In this study, growth phase-associated changes were investigated in *B. lactis* A6 based on RNA-seq analysis. The bacterium expanded the carbon source utilizing profile to transport cellobiose, XOS and raffinose in the stationary phase and tended to metabolize L-arabinose and xylose with reduced glycolysis in the stationary phase. Meanwhile, genes involved in acid tolerance response were upregulated, and *in vitro* experiment proved that the stationary phase cells are more acid-tolerant than the log phase cells. Remarkably, a putative gene cluster encoding Tad pili was significantly up-regulated at the stationary phase, accompanied with an enhanced bacterial adhesion to mucin at this stage. These findings provide a novel insight into the growth phase associated characteristics in *B. lactis* A6 and provide valuable information for further application of *B. lactis* A6 in the food industry.

## Methods

### Bacterial strains and culture conditions

*B. lactis* A6 was isolated from the feces of a centenarian in Bama, Guangxi, China and collected from the China General Microbiological Culture Collection Center (CGMCC) with CGMCC No. 9273. A -80°C glycerol stock of *B. lactis* A6 was anaerobically (100% N_2_) incubated at 37°C in MRSc broth for 24 h, followed by 1% (v/v) inoculation to a 15 mL MRSc for overnight. The basal MRSc broth contained the following sources (per liter of deionized water): 10 g of beef extract, 5 g of yeast extract, 10 g of tryptone peptone, 20 g of glucose, 2 g of triamine citrate, 2 g of K_2_HPO_4_, 0.5 g of MgSO_4_, 0.25 g of MnSO_4_, 5 g of sodium acetate, 1 mL of Tween-80 and 0.5 g of L-cysteine. The pH and optical density at 600 nm (OD_600_) were recorded at 2-h intervals.

### RNA extraction

Overnight culture of *B. lactis* A6 strain was anaerobically (100% N_2_) inoculated (1% v/v) into a 15 mL fresh MRSc broth and incubated at 37°C. Bacterial cells were harvested in the exponential phase (OD_600_ = 0.6-0.8) and the stationary phase (OD_600_ = 4.0-4.2) by centrifugation at 4°C, 8000 × g for 5 min. Total RNA was extracted using TRIzol reagent (Invitrogen, Carlsbad, CA) and purified using the TURBO DNA-free Kit (Ambion, Austin, TX, USA) according to the manufacturer’s instructions. RNA concentration was measured using Qubit® RNA Assay Kit in Qubit® 2.0 Flurometer (Life Technologies, CA, USA). RNA integrity was assessed using the RNA Nano 6000 Assay Kit of the Agilent Bioanalyzer 2100 system (Agilent Technologies, CA, USA).

### RNA-seq

Sequencing libraries were generated using NEBNext® Ultra™ Directional RNA Library Prep Kit for Illumina® (NEB, USA). Index codes were added to attribute sequences to each sample. The library fragments were purified with AMPure XP system (Beckman Coulter, Beverly, USA) to select cDNA fragments of 150~200 bp in length. Then 3 μl USER Enzyme (NEB, USA) was used with size-selected, adaptor-ligated cDNA at 37°C for 15 min followed by 95°C for 5 min. The PCR was performed using Phusion High-Fidelity DNA polymerase (NEB) with Universal PCR primers and Index (X) primer. Finally, PCR products were purified by AMPure XP system (Beckman Coulter) and the library quality was assessed on the Agilent Bioanalyzer 2100 system (Agilent Technologies). The library preparations were sequenced on an Illumina Hiseq platform and paired-end reads were generated.

### Transcriptomic data processing

Raw data of fastq format were firstly processed through in-house perl scripts. Clean reads were obtained by removing reads containing adapter, reads containing ploy-N or reads with low quality from raw data. All clean reads were aligned to the genome of *B. lactis* A6 with GenBank Accession No. NZ_CP010433.1 by Bowtie2-2.2.3 [47]. HTSeq v0.6.1 was then used to count the numbers of reads mapped to each gene and calculate the expected number of Fragments Per Kilobase of transcript sequence per Millions base pairs sequenced (FPKM) value [48]. Differential expression analysis of samples at the stationary growth phase compared with exponential phase was performed using the DESeq R package (V1.18.0) [49]. Genes with an adjusted *P*-value < 0.05 and |Fold change| ≥ 1.5 were assigned as differentially expressed. The metabolic pathways involved in these DEGs were further analyzed by KEGG enrichment analysis [50].

### Acid tolerance test

The acid tolerance of *B. lactis* A6 was performed according to Waddington et al. with some modifications [51]. Briefly, the bacteria were anaerobically (100% N_2_) incubated at 37°C in MRSc broth. Cells were harvested at the log growth phase (OD_600_ = 0.6-0.8) and the stationary phase (OD_600_ = 4.0-4.2). After centrifugation at 8000 × g, 22°C for 5 min, cell pellets were washed twice with 0.85% NaCl solution and adjusted the initial bacterial numbers to ~10^8^ CFU/mL. Aliquots of 1 mL cell suspension were collected and centrifugated at 8000 × g, 22°C for 5 min. The cell pellets were then suspended in 1 mL fresh MRSc broth at pH values of 4.0, 3.0, or 2.0 (adjusted by lactic acid). After anaerobic incubation at 37°C for 2 h, the bacterial number (CFU/mL) was determined by plating 10-fold serial dilutions on MRSc agar and incubating anaerobically at 37°C for 40 h. The bacterial cells untreated with acid were included as a control. The survival rate was calculated as follows: Survival rate (%) = [(CFU/mL) _acid treatment_ / (CFU/mL) _control_] × 100. The results were obtained by three independent experiments.

### Bacterial adhesion to mucin

The amino acid sequence alignment of Tad pili between *B. breve* UCC2003 and *B. lactis* A6 was performed with SnapGene software (from Insightful Science, version 5.3) in a local alignment (Smith-Waterman) model. The adherence of bacteria to mucin was performed as described by Xiong et al. [52]. Briefly, Nunc MaxiSorp 96-well microplate (Thermo Fisher Scientific, Denmark) was coated with 100 μL of mucin (from porcine stomach; Sigma-Aldrich, USA) and incubated for 16 h at 4°C at a concentration of 2.5 pmol per well. The wells were then washed twice with PBS and incubated with 2% (w/v) bovine serum albumin for 2 h at 37°C. The wells were subsequently washed three times with PBS. The bacteria collected at the exponential and stationary phase were washed twice with PBS and resuspended to a final OD_600_ equivalent to ~2-3 × 10^8^ CFU/mL. Then 200 μL of bacterial suspension were added to coated 96-well plates and incubated at 37°C for 1 h. The unattached bacteria were removed by washing the wells for 3 times with PBS, and then treated with 200 μL 0.05% (v/v) Triton X-100 and incubated at 37°C for 30 min. The adhesion ratios (%) were calculated by comparing the bacterial counts after adhesion to the number of cells originally added to the plate wells. The results were obtained by three independent experiments.

### Statistical analysis

Data were analyzed using GraphPad Prism 8 software for Windows (GraphPad Software, Inc., La Jolla, CA, USA). Results were presented as the mean value ± standard deviation. An unpaired Student t-test was used to calculate *P* values when two groups were compared.

## Supplementary Information

**Additional file 1.**

**Additional file 2.**

## Data Availability

All the raw data of transcriptome sequencing could be downloaded from GEO with the Accession No. GSE173957.

## Abbreviations

*B. lactis* A6: *Bifidobacterium animalis* subsp. *lactis* A6
*L. rhamnosus* GG: *Lactobacillus rhamnosus* GG
*L. casei* Zhang: *Lactobacillus casei* Zhang
*B. breve* UCC2003: *Bifidobacterium breve* UCC2003
RNA-seq: RNA-sequencing technology
MRSc: de Man-Rogosa-Sharp broth with L-cysteine
DEGs: Differential expression genes
GO: Gene ontology
KEGG: Kyoto Encyclopedia of Genes and Genomes
XOS: Xylooligosaccharides
GOS: Galactooligosaccharides
GlcN-6P: Glucosamine-6-phosphate
GlcN-1P: Glucosamine-1-phosphate
CCC: Cysteine-cystathionine-cycle
BCAAs: Branched-chain amino acids
Tad pili: Type IVb tight adherence pili
CGMCC: China General Microbiological Culture Collection Center
FPKM: Expected number of Fragments Per Kilobase of transcript sequence per Millions base pairs sequenced
SEG: Specifically expressed genes
LAB: Lactic acid bacteria
