# Supplementary material for: Transcriptome analysis revealed growth phase-associated changes of a centenarian-originated probiotic Bifidobacterium animalis subsp. lactis A6
Source: BMC Microbiol. 2022 Feb 25;22:61. doi: 10.1186/s12866-022-02474-5 (PMC8876546; doi:10.1186/s12866-022-02474-5)
Supplement: Supplementary file 2 — Additional file 2. [file 12866_2022_2474_MOESM2_ESM.docx]

*Supplementary Material*

**Transcriptome analysis revealed growth phase-associated changes of a centenarian-originated probiotic *Bifidobacterium animalis* subsp*. lactis* A6**

Hui Wang^1^, Jieran An^1^, Chengfei Fan^1^, Zhengyuan Zhai^1, 2^, Hongxing Zhang^3^, Yanling Hao^1, 2*^

^1^Key Laboratory of Functional Dairy, Co-constructed by Ministry of Education and Beijing Municipality, College of Food Science and Nutritional Engineering, China Agricultural University, Beijing, China

^2^Beijing Advanced Innovation Center for Food Nutrition and Human Health, College of Food Science and Nutritional Engineering, China Agricultural University, Beijing, China

^3^Department of Food Science, Beijing University of Agriculture, 7 Bei Nong Road, Changping District, Beijing 102206, China

**^*^Corresponding author:** Yanling Hao

**E-mail:** haoyl@cau.edu.cn

**Present address:** College of Food Science and Nutritional Engineering, China Agricultural University, 17 Qing Hua East Road, Hai Dian District, Beijing, 100083, China

**Table S1** The number of reads before and after the trimming step.

| Sample name | Raw reads | Clean reads | clean bases | Q20(%) | Q30(%) | GC content (%) |
| --- | --- | --- | --- | --- | --- | --- |
| A6log1 | 23311618 | 23261948 | 3.49G | 98.58 | 95.71 | 60.6 |
| A6log2 | 19689878 | 19652184 | 2.95G | 98.53 | 95.58 | 60.54 |
| A6log3 | 22553776 | 22508958 | 3.38G | 98.49 | 95.52 | 60.54 |
| A6WD2 | 23893390 | 23835722 | 3.58G | 98.29 | 94.88 | 59.87 |
| A6WD3 | 23501046 | 23447790 | 3.52G | 98.38 | 95.08 | 60.05 |
| A6WD4 | 21352442 | 21096818 | 3.16G | 98.15 | 94.5 | 60.75 |

**Table S2** The information of reads mapping to the reference genome.

| Sample name | A6log1 | A6log2 | A6log3 | A6WD2 | A6WD3 | A6WD4 |
| --- | --- | --- | --- | --- | --- | --- |
| Total reads | 23261948 | 19652184 | 22508958 | 23835722 | 23447790 | 21096818 |
| Total mapped | 23115123 (99.37%) | 19531490 (99.39%) | 22330874 (99.21%) | 23605032 (99.03%) | 23247112 (99.14%) | 20996723 (99.53%) |
| Multiple mapped | 464994  (2%) | 338173 (1.72%) | 447809 (1.99%) | 642864  (2.7%) | 585452  (2.5%) | 309065 (1.46%) |
| Uniquely mapped | 22650129 (97.37%) | 19193317 (97.67%) | 21883065 (97.22%) | 22962168 (96.34%) | 22661660 (96.65%) | 20687658 (98.06%) |

**Table S3** Specifically expressed genes in the stationary phase or in the log phase.

| **Group ^a^** | **Gene_ID ^b^** | **Function** |
| --- | --- | --- |
| **A6_WD** | BAA6_0047 | ISL3 family transposase |
|  | BAA6_0209 |  |
|  | BAA6_0239 | ISL3 family transposase |
|  | BAA6_0561 |  |
|  | BAA6_1129 | ISL3 family transposase |
|  | BAA6_1181 | helix-turn-helix domain-containing protein |
|  | BAA6_1182 | helix-turn-helix domain-containing protein |
|  | BAA6_1183 | hypothetical protein |
|  | BAA6_1185 | hypothetical protein |
|  | BAA6_1186 | AAA family ATPase |
|  | BAA6_1187 | hypothetical protein |
|  | BAA6_1188 | hypothetical protein |
|  | BAA6_1189 | hypothetical protein |
|  | BAA6_1190 | hypothetical protein |
|  | BAA6_1473 |  |
|  | Novel00011 | hypothetical protein |
|  | Novel00013 | hypothetical protein |
|  | Novel00017 | hypothetical protein |
| **A6_log** | BAA6_0096 | hypothetical protein |

^a^ A6_WD: stationary phase, A6_log: log phase.

^b^ Novel00011, Novel00013, and Novel00017 replicate novel transcripts annotated by Rockhopper software.

**Table S4** Genes differentially expressed at the transcriptional level in *B. lactis* A6 during the stationary phase compared to the exponential phase **^a^**.

| **Gene_ID** | **Gene** | | **Readcount_WD** | **Readcount_ log** | | | **padj ^b^** | | **Fold Change ^c^** | **Description ^d^** | | | |  |  |  |
| --- | --- | --- | --- | --- | --- | --- | --- | --- | --- | --- | --- | --- | --- | --- | --- | --- |
| **Carbohydrate transport and metabolism** | | | | | | | | | | | | | | | | |
| BAA6_0488 | |  | 4837.163 | | 11676.14 | 0.006421 | | -2.41 | | sugar ABC transporter substrate-binding protein | | | | |  |  |
| BAA6_0490 | |  | 756.8667 | | 2181.382 | 2.54E-06 | | -2.88 | | beta-galactosidase | | | | |  |  |
| BAA6_0491 | |  | 258.41 | | 564.07 | 0.0092014 | | -2.18 | | sugar ABC transporter permease | | | | |  |  |
| BAA6_1555 | |  | 1119.46 | | 2888.72 | 0.0000347 | | -2.58 | | MalG-type ABC sugar transport system permease component | | | | |  |  |
| BAA6_1552 | |  | 687.6769 | | 355.9917 | 0.042596 | | 1.93 | | alpha-amylase | | | | |  |  |
| BAA6_1553 | |  | 2280.263 | | 1091.734 | 9.64E-05 | | 2.09 | | 4-alpha-glucanotransferase | | | | |  |  |
| BAA6_1548 | |  | 4025.94 | | 7369.93 | 0.0024102 | | -1.83 | | putative sugar ABC transport system membrane protein | | | | |  |  |
| BAA6_1549 | |  | 5952.68 | | 12644.90 | 0.0013191 | | -2.12 | | putative sugar ABC transport system membrane protein | | | | |  |  |
| BAA6_0517 | |  | 2292.087 | | 893.0124 | 1.12E-06 | | 2.57 | | putative sugar transporter solute-binding protein | | | | |  |  |
| BAA6_0518 | |  | 489.5555 | | 225.4359 | 0.011554 | | 2.17 | | putative binding protein-dependent transporter | | | | |  |  |
| BAA6_1585 | |  | 373.3087 | | 115.6564 | 0.032655 | | 3.23 | | MalG-type ABC sugar transport system permease component | | | | |  |  |
| BAA6_1587 | |  | 561.3921 | | 206.0775 | 0.045279 | | 2.72 | | sugar binding protein of ABC transporter system | | | | |  |  |
| BAA6_0481 | |  | 272.5421 | | 43.66605 | 0.002365 | | 6.24 | | galactoside symporter【sodium:solute symporter】 | | | | |  |  |
| BAA6_0482 | | *lacZ* | 839.1074 | | 87.53825 | 0.0073465 | | 9.59 | | beta-galactosidase | | | | |  |  |
| BAA6_1092 | |  | 1164.061 | | 7143.124 | 1.10E-25 | | -6.14 | | glucose transporter | | | | |  |  |
| BAA6_0050 | |  | 1456.221 | | 406.8645 | 0.0001 | | 3.58 | | MFS transporter [gentiobiose transport] | | | | |  |  |
| BAA6_1415 | |  | 138.0016 | | 38.09751 | 0.014535 | | 3.62 | | carbohydrate ABC transporter permease | | | | |  |  |
| BAA6_1416 | |  | 351.0663 | | 45.18199 | 0.001189 | | 7.77 | | cellobiose phosphorylase | | | | |  |  |
| BAA6_0431 | | *pgM* | 12958.58 | | 19800.90 | 0.013612 | | -1.53 | | phosphoglucomutase | | | | |  |  |
| BAA6_0298 | | *gpi* | 38832.89 | | 61247.24 | 0.009699 | | -1.58 | | glucose-6-phosphate isomerase | | | | |  |  |
| BAA6_0968 | | *xfp* | 111945.49 | | 191409.80 | 0.0080577 | | -1.71 | | Xylulose-5-phosphate/fructose-6-phosphate phosphoketolase | | | | |  |  |
| BAA6_0838 | | *tkt* | 39744.28 | | 73805.30 | 0.0081896 | | -1.86 | | transketolase | | | | |  |  |
| BAA6_0839 | | *tal* | 30636.18 | | 61463.04 | 0.00012407 | | -2.01 | | transaldolase | | | | |  |  |
| BAA6_0794 | | *rpe* | 2347.70 | | 3849.54 | 0.0033913 | | -1.64 | | ribulose-phosphate 3-epimerase | | | | |  |  |
| BAA6_0846 | |  | 3950.11 | | 9579.81 | 2.0466E-08 | | -2.43 | | triosephosphate isomerase | | | | |  |  |
| BAA6_1161 | | *gadph* | 39507.34 | | 63632.15 | 0.012168 | | -1.60 | | Glyceraldehyde-3-phosphate dehydrogenase | | | | |  |  |
| BAA6_0847 | | *pgk* | 34130.46 | | 58045.92 | 0.001297 | | -1.70 | | phosphoglycerate kinase | | | | |  |  |
| BAA6_0756 | | *pgm* | 2448.33 | | 6422.61 | 0.00002579 | | -2.62 | | phosphoglycerate mutase | | | | |  |  |
| BAA6_0939 | |  | 952.61 | | 2138.74 | 0.00003033 | | -2.25 | | phosphoglycerate mutase | | | | |  |  |
| BAA6_1012 | | *eno* | 28742.54 | | 48573.96 | 0.0081868 | | -1.69 | | enolase | | | | |  |  |
| BAA6_0979 | | *pyk* | 24139.44 | | 36961.71 | 0.003455 | | -1.53 | | pyruvate kinase | | | | |  |  |
| BAA6_0329 | | *ldh2* | 1179.00 | | 3090.27 | 6.2106E-08 | | -2.62 | | lactate dehydrogenase | | | | |  |  |
| BAA6_1078 | | *pfl* | 27279.84 | | 98480.85 | 3.2808E-09 | | -3.61 | | formate acetyltransferase | | | | |  |  |
| BAA6_0969 | | *pat* | 2129.85 | | 7041.85 | 3.4426E-13 | | -3.31 | | phosphate acetyltransferase | | | | |  |  |
| BAA6_0970 | | *ackA* | 9690.04 | | 16608.64 | 0.00033404 | | -1.71 | | acetate kinase | | | | |  |  |
| BAA6_0061 | |  | 12976.47 | | 6841.51 | 0.0018613 | | 1.90 | | alpha-L-arabinofuranosidase | | | | |  |  |
| BAA6_0062 | | *araB* | 3423.84 | | 907.13 | 2.2266E-06 | | 3.77 | | L-ribulokinase | | | | |  |  |
| BAA6_0063 | | *araD* | 3484.27 | | 790.10 | 0.00037944 | | 4.41 | | L-ribulose-5-phosphate 4-epimerase | | | | |  |  |
| BAA6_0064 | | *araA* | 6100.34 | | 1776.17 | 0.009295 | | 3.43 | | L-arabinose isomerase | | | | |  |  |
| BAA6_0525 | | *xylB* | 577.37 | | 278.73 | 0.0060988 | | 2.07 | | xylulose kinase | | | | |  |  |
| BAA6_1489 | |  | 1152.05 | | 2816.30 | 9.9091E-07 | | -2.44 | | ribose kinase | | | | |  |  |
| BAA6_1351 | | *galK* | 1998.56 | | 5957.44 | 2.8904E-11 | | -2.98 | | galactokinase | | | | |  |  |
| BAA6_1352 | | *galT* | 2187.794 | | 4623.092 | 4.60E-06 | | -2.11 | | galactose-1-phosphate uridylyltransferase | | | | |  |  |
| BAA6_0523 | | *xyn* | 1098.73 | | 436.21 | 0.0041908 | | 2.52 | | endo-1,4-β-xylanase | | | | |  |  |
| **Amino acids uptake and biosynthesis** | | | | | | | | | | | | | | | |  |
| BAA6_0566 | | *oppA* | 15062.53 | | 2875.62 | 1.623E-11 | | 5.24 | | oligopeptide transporter substrate-binding protein | | |  |  |  |  |
| BAA6_0567 | | *oppB* | 1772.75 | | 850.70 | 0.00061858 | | 2.08 | | ABC transporter, oligopeptide transporter permease component | | |  |  |  |  |
| BAA6_0568 | | *oppC* | 967.68 | | 474.69 | 0.028271 | | 2.04 | | ABC transporter, oligopeptide transporter permease component | | |  |  |  |  |
| BAA6_0570 | | *oppF* | 1462.26 | | 533.70 | 2.7866E-06 | | 2.74 | | ABC transporter, oligopeptide transporter ATPase component | | |  |  |  |  |
| BAA6_1193 | | *dppD* | 349.55 | | 41.42 | 0.0035088 | | 8.44 | | ABC transporter, dipeptide transporter ATPase component | | |  |  |  |  |
| BAA6_1194 | | *dppC* | 191.84 | | 32.96 | 0.029065 | | 5.82 | | ABC transporter, dipeptide transporter permase component | | |  |  |  |  |
| BAA6_1269 | |  | 1827.44 | | 6028.06 | 1.4064E-09 | | -3.30 | | peptide ABC transporter permease | | |  |  |  |  |
| BAA6_1270 | |  | 806.05 | | 1634.59 | 0.0005981 | | -2.03 | | ATP binding protein of ABC transporter for Glu/Asp | | |  |  |  |  |
| BAA6_0681 | | *gluB* | 1159.61 | | 2635.49 | 8.9215E-06 | | -2.27 | | ABC-type amino acid transport system periplasmic component | | |  |  |  |  |
| BAA6_0682 | | *gluC* | 459.24 | | 1351.74 | 5.918E-07 | | -2.94 | | ABC-type amino acid transport system permease component | | |  |  |  |  |
| BAA6_0683 | | *gluD* | 1224.26 | | 3299.96 | 8.0742E-08 | | -2.70 | | permease protein of ABC transporter for glutamate | | |  |  |  |  |
| BAA6_0680 | | *gluA* | 2357.32 | | 5816.65 | 9.8725E-08 | | -2.47 | | ATP-binding protein of ABC transporter for glutamate | | |  |  |  |  |
| BAA6_1082 | | *MetI* | 406.44 | | 1817.56 | 3.2421E-13 | | -4.47 | | ABC-type amino acid transport system permease component | | |  |  |  |  |
| BAA6_1083 | | *MetN* | 3224.53 | | 6827.91 | 5.8124E-06 | | -2.12 | | ATP binding protein of ABC transporter | | |  |  |  |  |
| BAA6_0186 | |  | 4483.57 | | 2664.15 | 0.0036456 | | 1.68 | | dipeptidase | | |  |  |  |  |
| BAA6_0230 | |  | 4268.44 | | 2137.39 | 0.00007977 | | 2.00 | | dipeptidase | | |  |  |  |  |
| BAA6_0261 | | *aspC* | 3228.57 | | 5435.42 | 0.0024363 | | -1.68 | | aspartate aminotransferase [aspartate biosynthesis] | | |  |  |  |  |
| BAA6_0881 | | *metE* | 2053.52 | | 3229.828 | 0.035923 | | -1.57 | | homocysteine methyltransferase [methionine biosynthesis] | | |  |  |  |  |
| BAA6_1150 | | *pheA* | 856.79 | | 1947.80 | 0.00001933 | | -2.27 | | bifunctional chorismate mutase/prephenate dehydratase  [tyrosine and phenylalanine biosynthesis] | | |  |  |  |  |
| BAA6_0731 | | *argF* | 1973.50 | | 9750.90 | 5.1355E-23 | | -4.94 | | ornithine carbamoyltransferase [arginine biosynthesis] | | |  |  |  |  |
| BAA6_0733 | | *argG* | 2812.10 | | 45962.99 | 3.5605E-65 | | -16.35 | | argininosuccinate synthase [arginine biosynthesis] | | |  |  |  |  |
| BAA6_0734 | | *argH* | 4192.08 | | 20956.55 | 8.4638E-25 | | -5.00 | | argininosuccinate lyase [arginine biosynthesis] | | |  |  |  |  |
| BAA6_1052 | | *proC* | 780.68 | | 2597.57 | 2.3774E-10 | | -3.33 | | pyrroline-5-carboxylate reductase [proline biosynthesis] | | |  |  |  |  |
| BAA6_1220 | | *glnA* | 2812.383 | | 6147.336 | 1.70E-06 | | -2.19 | | glutamine synthetase [glutamine biosynthesis] | | |  |  |  |  |
| **Peptidoglycan biosynthesis** | | | | | | | | | | | | | | | | |
| BAA6_0562 | | *glmS* | 9141.46 | | 19718.33 | 1.83E-07 | | -2.16 | | D-fructose-6-phosphate amidotransferase |  |  |  |  |  |  |
| BAA6_1325 | | *glmM* | 6252.09 | | 11701.55 | 0.00005026 | | -1.87 | | phosphoglucosamine mutase |  |  |  |  |  |  |
| BAA6_0751 | | *glmU* | 2120.94 | | 6535.18 | 6.3788E-12 | | -3.08 | | bifunctional protein glmU |  |  |  |  |  |  |
| BAA6_1199 | | *murC* | 1762.37 | | 4737.43 | 3.6581E-09 | | -2.69 | | UDP-N-acetylmuramate--L-alanine ligase |  |  |  |  |  |  |
| BAA6_1202 | | *murD* | 3254.93 | | 6423.39 | 0.00002404 | | -1.97 | | UDP-N-acetylmuramoyl-L-alanine-D-glutamate ligase |  |  |  |  |  |  |
| BAA6_1204 | | *murF* | 3877.04 | | 6141.24 | 0.003391 | | -1.58 | | UDP-N-acetylmuramoyl-tripeptide--D-alanyl-D- alanine ligase |  |  |  |  |  |  |
| BAA6_1612 | | *murJ* | 8799.44 | | 14272.57 | 0.001764 | | -1.62 | | putative peptidoglycan lipid II flippase |  |  |  |  |  |  |
| BAA6_0083 | |  | 3520.72 | | 5666.97 | 0.012753 | | -1.61 | | transpeptidase |  |  |  |  |  |  |
| BAA6_0084 | |  | 2980.97 | | 6028.54 | 0.00050261 | | -2.02 | | bacterial cell division membrane protein |  |  |  |  |  |  |
| BAA6_0550 | | *ftsQ* | 1417.70 | | 5084.21 | 6.9834E-14 | | -3.59 | | cell division protein FtsQ |  |  |  |  |  |  |
| BAA6_1198 | | *ftsQ* | 1336.00 | | 3695.44 | 5.5601E-09 | | -2.77 | | cell division protein FtsQ |  |  |  |  |  |  |
| BAA6_1201 | | *ftsW* | 1863.09 | | 5417.81 | 1.5629E-10 | | -2.91 | | cell division protein FtsW |  |  |  |  |  |  |
| **Acid tolerance response** | | | | | | | | | | | | | | | | |
| BAA6_0643 | | *serB* | 1899.74 | | 1074.17 | 0.0027692 | | 1.77 | | phosphoserine phosphatase SerB [serine biosynthesis] | |  |  |  |  |  |
| BAA6_0572 | | *cysK* | 2229.86 | | 314.94 | 0.00002042 | | 7.08 | | cystathionine beta-synthase | |  |  |  |  |  |
| BAA6_0947 | | *metC* | 263.30 | | 93.88 | 0.0075693 | | 2.80 | | cystathionine beta-lyase | |  |  |  |  |  |
| BAA6_0010 | | *gdhA* | 37283.59 | | 11567.72 | 0.0036456 | | 3.22 | | glutamate dehydrogenase | |  |  |  |  |  |
| BAA6_0708 | | *glnA1* | 2834.66 | | 21021.62 | 1.706E-08 | | -7.42 | | glutamine synthetase 1 [glutamine biosynthesis] | |  |  |  |  |  |
| BAA6_1220 | | *glnA2* | 2812.38 | | 6147.34 | 1.6962E-06 | | -2.19 | | glutamine synthetase 2 [glutamine biosynthesis] | |  |  |  |  |  |
| BAA6_0174 | | *livK* | 489.16 | | 160.08 | 0.013237 | | 3.06 | | BCAAs transport system substrate-binding protein | |  |  |  |  |  |
| BAA6_0283 | | *ilvB* | 12471.77 | | 4811.92 | 8.2728E-08 | | 2.59 | | acetolactate synthase 1 catalytic subunit | |  |  |  |  |  |
| BAA6_0146 | | *ilvC* | 115764.63 | | 28148.96 | 1.3484E-09 | | 4.11 | | ketol-acid reductoisomerase [isoleucine biosynthesis] | |  |  |  |  |  |
| BAA6_0675 | | *clpE* | 17230.05 | | 10655.04 | 0.002340 | | 1.62 | | ATP-dependent Clp protease ATP-binding subunit | |  |  |  |  |  |
| BAA6_1072 | | *clpP1* | 4511.64 | | 2832.18 | 0.009528 | | 1.59 | | ATP-dependent Clp protease proteolytic subunit | |  |  |  |  |  |
| BAA6_1073 | | *clpP2* | 6299.84 | | 1905.59 | 8.5363E-13 | | 3.31 | | ATP-dependent Clp protease proteolytic subunit 2 | |  |  |  |  |  |
| **Adhesion** | | | | | | | | | | | | | | | | |
| BAA6_0205 | | *tadZ* | 610.44 | | 58.87 | 0.0012024 | | 10.37 | | Flp pilus assembly protein tadZ | |  |  |  |  |  |
| BAA6_0206 | | *tadA* | 355.34 | | 50.65 | 0.0022085 | | 7.02 | | Flp pilus assembly protein ATPase component | |  |  |  |  |  |
| BAA6_0207 | | *tadB* | 314.23 | | 25.36 | 0.0035007 | | 12.39 | | Flp pilus assembly protein | |  |  |  |  |  |
| BAA6_0208 | | *tadC* | 142.71 | | 13.50 | 0.028494 | | 10.57 | | Flp pilus assembly protein | |  |  |  |  |  |
| BAA6_0210 | | *flp* | 34.58 | | 4.58 | 0.038771 | | 7.54 | | fimbrial protein prepilin | |  |  |  |  |  |
| BAA6_0211 | | *tadE* | 172.20 | | 22.28 | 0.0060286 | | 7.73 | | pseudopilin TadE | |  |  |  |  |  |
| BAA6_0212 | | *tadF* | 321.74 | | 40.04 | 0.00080135 | | 8.04 | | pseudopilin TadF | |  |  |  |  |  |
| BAA6_0224 | | *tadV* | 6055.33 | | 3936.56 | 0.0089983 | | 1.54 | | prepilin peptidase | |  |  |  |  |  |

^a^ Selected genes which were discussed in the Results section.

^b^ Adjusted p-value based on false discovery rate (FDR) value of multiple hypothesis testing.

^c^ Fold changes of differential expressed genes in the stationary phase (WD) compared to the exponential phase (log). The minus number means down-regulated genes.

^d^ Functions were assigned from the KEGG pathways for *B.* *lactis* A6.

**Table S5** Alignment of amino acid sequences of tad pili in *B. lactis* A6 compared with *B. breve* UCC2003 by SnapGene software (version 5.3) in a local alignment (Smith-Waterman) module.

| Gene number | | Gene name | BLAST results | | | |
| --- | --- | --- | --- | --- | --- | --- |
| A6 | UCC2003 |  | length | Identity | Similarity | Gap |
| BAA6_0205 | Bbr_0132 | *tadZ* | 280 | 38.93% | 56.43% | 11.07% |
| BAA6_0206 | Bbr_0133 | *tadA* | 327 | 59.33% | 70.03% | 0.00% |
| BAA6_0207 | Bbr_0134 | *tadB* | 206 | 39.32% | 48.06% | 16.02% |
| BAA6_0208 | Bbr_0135 | *tadC* | 168 | 41.07% | 57.14% | 17.86% |
| BAA6_0210 | Bbr_0136 | *flp* | 84 | 58.33% | 76.19% | 4.76% |
| BAA6_0211 | Bbr_0137 | *tadE* | 103 | 38.83% | 51.46% | 0.00% |
| BAA6_0212 | Bbr_0138 | *tadF* | 117 | 34.19% | 52.99% | 5.93% |
| BAA6_0933 | Bbr_0901 | *tadV* | 159 | 36.48% | 55.97% | 5.03% |
| BAA6_0224 | Bbr_0901 | *tadV* | 135 | 25.19% | 37.78% | 5.93% |


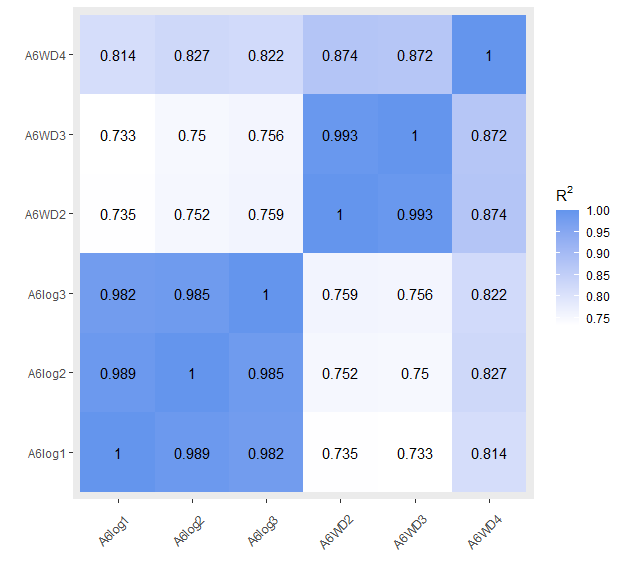


**Fig. S1.** Pearson correlation between samples. Three replicates were analyzed for each growth phase: A6_log1, A6_log2, and A6_log3 for exponential phase; A6_WD2, A6_WD3, and A6_WD4 for stationary phase.


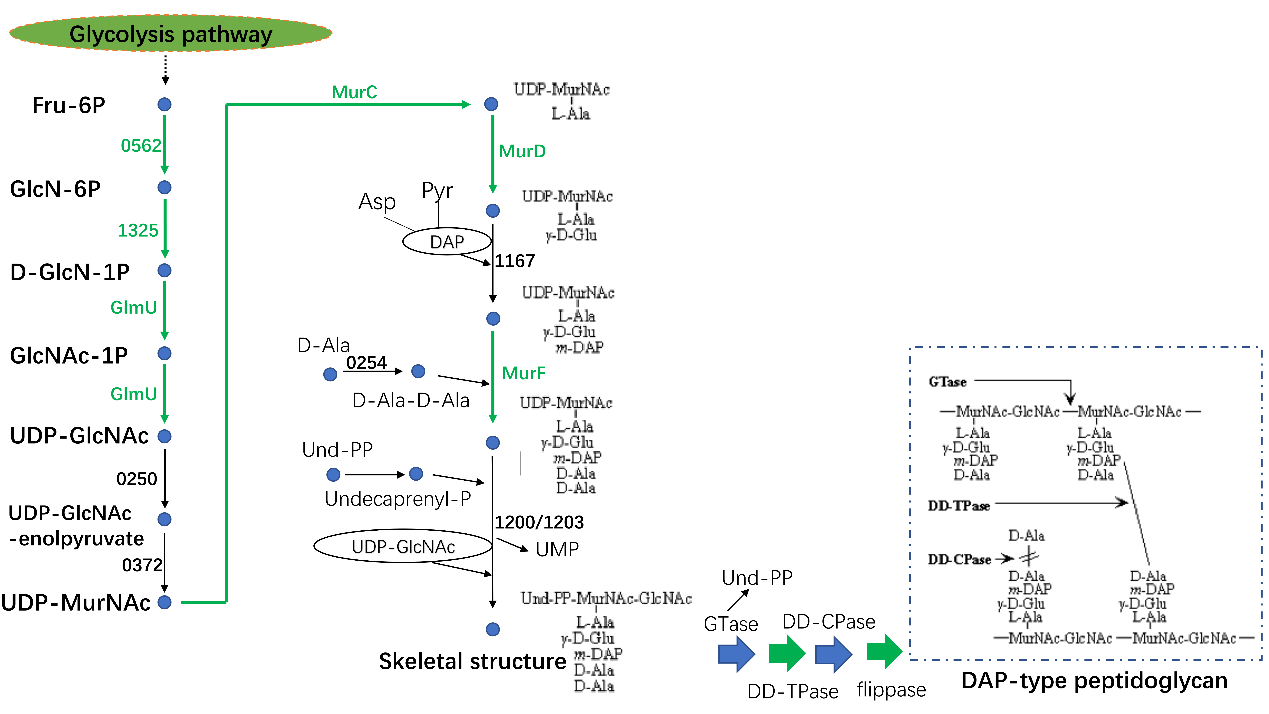


**Fig. S2.** Graphical representation of DAP-type peptidoglycan biosynthesis of *B. lactis* A6 in the stationary phase compared to the log phase. Fru-6P, fructose-6-phosphate; GlcN-6P, glucosamine-6-phosphate; D-GlcN-1P, glucosamine-1-phosphate; GlcNAc-1P, N-acetylglucosamine-1-phosphate; UDP-GlcNAc, uridine diphosphate-N-acetylglucos-amine; UDP-MurNAc, UDP-N-acetylmuramate; Und-PP, undercaprenyl diphosphate; GlmU, N-acetylglucosamine-1-phosphate uridyltransferase; MurC, UDP-N-acetylmuramate L-alanine ligase; MurD, UDP-N-acetylmuramoylalanine D-glutamate ligase; MurF, UDP-N-acetylmuramoyl-tripeptide D-alanyl-D-alanine ligase. Arrows and characters in black and green color represent genes which were un-changed and down-regulated, respectively. The peptidoglycan biosynthesis pathway and structural formulas are referenced from the KEGG pathway database (<https://www.genome.jp/pathway/map00550>) [1].

Reference:

[1] Kanehisa M, and Goto S. KEGG: kyoto encyclopedia of genes and genomes. Nucleic Acids Res. 2000, 28, 27-30.
